# Low-intensity pulsed ultrasound ameliorates partial infraorbital nerve ligation-induced trigeminal neuropathic pain through inhibiting Schwann cell Pannexin 1 channel

**DOI:** 10.3389/fimmu.2025.1712759

**Published:** 2026-01-05

**Authors:** Yao Liu, Xue Han, Weijie Zhang, Qingguo Pei, Shuyu Xu, Haicheng Wang

**Affiliations:** 1 Department of Implantology, Stomatological Hospital and Dental School of Tongji University, Shanghai Engineering Research Center of Tooth Restoration and Regeneration, Shanghai, China; 2 Department of Pediatric Dentistry, Stomatological Hospital and Dental School of Tongji University, Shanghai Engineering Research Center of Tooth Restoration and Regeneration, Shanghai, China; 3 Department of Stomatology, Shanghai General Hospital, Shanghai Jiao Tong University School of Medicine, Shanghai, China

**Keywords:** nociceptive pain, glial cell, trigeminal ganglion, ultrasonic therapy, immunity, neuroinflammation

## Abstract

**Background:**

Neuroinflammation significantly contributes to trigeminal neuropathic pain (TNP). Low-intensity pulsed ultrasound (LIPUS) showed anti-inflammatory function in several diseases. It is still unknown that whether LIPUS show its analgesic effect against TNP. This study investigated how LIPUS alleviates pain in mice with TNP from partial infraorbital nerve ligation (pIONL).

**Materials and Methods:**

ICR mice, 7–11 weeks, were prepared. von Frey test was used to analyze all the nocifensive behavior score. RNA-sequencing was performed on the infraorbital nerve (ION) three days post-pIONL and on 24-hour cultured Schwann cells to identify inflammation-related genes and pathways. RT-qPCR, western blotting, immunofluorescent staining was used to analyze the expressions of Pannexin 1 channel and pro-inflammatory cytokines *in vivo* and *in vitro* studies.

**Results:**

pIONL induced persistent neuroinflammatory responses and mechanical allodynia, which were ameliorated by LIPUS treatment. Panx 1 was highly expressed after pIONL., LIPUS treatment inhibited pIONL-induced neuroinflammation in ION, trigeminal ganglion, and spinal cord tissue. Inhibition of Panx 1 via siRNA significantly attenuated the mechanical allodynia. Several cytokines were inhibited by Panx 1-siRNA, both Panx 1-siRNA and LIPUS treatment suppressed several cytokines. Next, we cultured Schwann cells with TNF-α (200ng/ml). We found LIPUS effectively downregulated the expression of Panx 1 and pro-inflammatory cytokines *in Vitro*. Intra-ION injection of BzATP induced TNP, which was ameliorated by LIPUS along with downregulation of Panx 1 and pro-inflammatory cytokines,. RNA-sequencing analysis revealed that LIPUS downregulates pathways related to inflammation, ion channels, and metabolism in Schwann cells.

**Conclusions:**

This study demonstrates that LIPUS exerts an analgesic effect by targeting Panx1 in Schwann cells of the peripheral nervous system, thereby ameliorating neuroinflammation and providing sustained relief from TNP.

## Introduction

Trigeminal neuropathic pain (TNP) is a severe pain in the trigeminal nerve region, which is defined by spontaneous and elicited paroxysms of electric shock-like or stabbing pain. TNP most frequently affects mandibular or maxillary division of the trigeminal nerve. The incidence of TNP is much higher in women than in men and increases with age (1).

The complex pathogenesis of nerve injury induced TNP makes clinical management challenging.Studies indicate that neuroinflammation contributes to neuropathic pain development, including TNP. Previous studies focused on neuronal cells, such as primary sensory neurons. However, recent researchers suggest that glial cells—which are non-neuronal cells—play a significant part in the pathophysiology of neuropathic pain (NP). The balance between excitatory and inhibitory synaptic transmission in the central nervous system (CNS) is crucial for pain inhibition (2–4). Previous studies reported that glial cells in CNS play crucial roles in pain modulation. However, growing evidence pay attention to Schwann cells in peripheral system. Schwann cells release numerous signaling molecules, these molecules interact with the long axons. Following peripheral nerve damage, Schwann cells activate and produce a variety of active chemicals. Multiple channels and receptors are activated. Substantial evidence underscores Schwann cells’ pivotal role in modulating NP (3, 5, 6).

Pannexins (Panx) form high-porosity membrane channels that are highly permeable to ATP and various signaling molecules. In the nervous system, Panx 1 located in neurons, glial cells and associated with development of neuropathic pain in microglia and astrocytes in CNS and dorsal root ganglion (7–9). Previous studies revealed that Schwann cells highly express Panx 1 compared to Panx 2 and Panx 3 (10), we focused on Panx 1 due to its high expression in Schwann cells and established role in neuroinflammation. Also, a recent study has offered a fresh perspective that Schwann cells Panx 1 modulated chronic constriction injury induced neuropathic pain by modulating neuroinflammation (11). However, the role of Schwann cell Panx 1 in TNP remains unclear.

Low-intensity pulsed ultrasound (LIPUS) is a therapeutic, diagnostic, non-surgical, safe, and potentially useful tool in the clinic. LIPUS has shown the efficacy of promoting spinal fusion by regulating M1-polarized macrophage to M2-polarized macrophage (12). Also, LIPUS accelerated the regeneration of sciatic nerve (13). Meanwhile, LIPUS has no adverse effects, while we used standardized parameters (1.5 MHz, 30 mW/cm²), future studies should optimize LIPUS settings (e.g., frequency, intensity) for maximal efficacy in TNP. Research indicates LIPUS promotes tissue repair and reduces inflammation across multiple conditions, including skeletal muscle damage, sialadenitis, and knee joint synovitis (14–16). We also reported LIPUS ameliorates partial sciatic nerve ligation-induced neuropathic pain by inducing M2-polarized macrophages (17). However, the anti-TNP activity of LIPUS and the role of LIPUS on activated Schwann cells have not been investigated.

Herein, we investigated the analgesic role of LIPUS against TNP induced by partial infraorbital nerve ligation (pIONL) and potential mechanisms.

## Materials and methods

### Animals

The animal research was approved by the Animal Research Committee of Stomatological Hospital and Dental School of Tongji University ((2022)-SR-06). All animal studies adhered to IASP ethical guidelines (18). Male ICR mice (ICR, Leigen company) aged 7–11 weeks were randomly assigned to experimental groups, – Male mice were used to minimize variability due to hormonal cycles, which is common in neuropathic pain studies. Rodents were kept in standard lab conditions (12-hour light/dark schedule) with unrestricted access to water and nourishment. All work has been reported according to the ARRIVE criteria (19).

### pIONL ligation, LIPUS exposure and regent injection

pIONL was performed according to a previous study to induce TNP (20, 21). Mice were deeply anesthetized with sodium pentobarbital and with an intraperitoneal injection (40 mg/kg). Right infraorbital nerve (ION) was exposed. An incision around 1mm was established in front of tendon. ION was divided using a glass rod, followed by the prompt ligation of the nerve with 8–0 silk thread, surgical success was confirmed by post-operative mechanical allodynia development.

Following the pIONL protocol, the LIPUS device (Osteotron V, ITO Co., Tokyo, Japan) was utilized on the injured whisker pad with a frequency of four or eight treatments, spanning days 0 to 3 and days 0 to 7. A sterile ultrasound gel was used as a coupling medium between the transducer and the skin to ensure efficient energy transfer. LIPUS was applied at 1.5 MHz, spatial average temporal average intensity of 30 mW/cm², duty cycle of 20%, pulse repetition frequency of 1.5kHz, and pulse width of 200 μs. The transducer had a beam non-uniformity ratio of 3.6,, and used for 20 minutes daily. Parameters were chosen based on previous studies showing efficacy in neuropathic pain (17). The circular transducer boasts a cross-sectional area of 5.0 cm², while the ultrasonic head’s active emitting surface measures 4.1 cm², and the beam’s uniformity ratio is maintained at 3.6.

Panx 1-siRNA (100μM) (5′-GCCTCATTAACCTCATTGT-3′) or NC-siRNA was diluted with sterile and nuclease free PBS, after that immediately used in the experiments. NC-siRNA was used as a negative control. Effective knockdown was validated by qPCR and Western blot. BzATP (200μM), which is agonist of P2X7 receptor, was diluted with DEPC water. The regent was immediately injected into ION after pIONL with a 29G syringe (Becton, Dickinson and Co, USA).

### Facial pain behavioral test

Before the initial round of assessments, the rodents underwent a 30-minute acclimatization process daily for two to three days, maintaining an environment with temperatures between 22 and 24 degrees Celsius and humidity levels between 40% and 60%. To evaluate the development of pIONL-induced allodynia, von Frey test was applied. Behavioral tests were conducted by experimenters blinded to group assignments, and data were coded until analysis. Mechanical allodynia was assessed using von Frey filaments (0.02 g and 0.16 g) applied to the whisker pad. Experimenters were blinded to group assignments. Withdrawal responses were recorded over five trials and scored as follows: 5 = face wiping >3 times; 4 = face wiping ≤3 times; 3 = rapid, severe paw withdrawal; 2 = mild withdrawal/face turning away; 1 = detection; 0 = no response.

### Real-time quantitative polymerase chain reaction and western blotting

On day 3, a fresh ION segment (0.5 cm) from the ligation site and cultured Schwann cell total RNA were collected; trigeminal ganglion and spinal cord samples were collected on day 7 using Isogen (Takara, Tokyo, Japan). RNA quality/quantity was assessed using a Nanodrop spectrophotometer (Thermo Scientific, USA). cDNA was synthesized using 1st strand cDNA synthesis supermix (Yeason, China). Primer sequences are listed in **Table 1**.

Tissues and cells were lysed with RIPA buffer (Sigma-Aldrich, USA) containing protease/phosphatase inhibitors. Proteins were separated by 10% SDS-PAGE, transferred to PVDF membranes, blocked with 5% skim milk in TBST, and incubated overnight at 4°C with primary antibodies against Panx1, TNF-α, IL-1β, and IL-6 (Boster). Membranes were then incubated with HRP-conjugated secondary antibodies, and signals were detected using a GE ImageQuant LAS system.

### Immunofluorescent staining

Mice were thoroughly sedated and then given 4% paraformaldehyde after intracardiac perfusion with PBS. For tissue examination, the gathered ION, TG, and C1–C2 spinal cord were utilized. The samples were treated with 4% paraformaldehyde from Nacalai Tesque in Kyoto, Japan, for a full day at 4 degrees Celsius, then carefully embedded in paraffin from Sakura Finetek Japan in Tokyo.

The samples were then precision-cut into 5-µm slices using a Thermo Fisher Scientific HM 450 Sliding Microtome. Next, they underwent a graded alcohol series—two rounds of 100%, followed by 90%, 80%, and 70% ethanol (Japan Alcohol Trading, Tokyo)—each step lasting five minutes. Deparaffinization and hydration were achieved through three five-minute xylene washes (Fujifilm Wako, Cat. No. 244-00081). For antigen retrieval, tissue sections were placed in a retrieval container with either citric acid buffer (pH 6.0) or EDTA-based alkaline solution (pH 9.0), then microwaved at medium power for 8 minutes, rested for another 8 minutes, and finally heated at medium-low for 7 minutes.

After cooling naturally, the slides were transferred to PBS (pH 7.4) and agitated in three five-minute cycles using a decolorizing shaker. Finally, sections were treated with 3% hydrogen peroxide in a dark environment for 15 minutes at room temperature to block endogenous peroxidase activity. After being submerged in PBS (PH7.4), the slides were shaken three times for five minutes each using a decolorization shaker. Subsequent to the partial desiccation of the sections, a super pap pen was employed to delineate a circle around the tissue. A 3% BSA-PBS solution was then introduced to uniformly saturate the area, followed by a blocking period at room temperature for 30 minutes. The sections were incubated with following primary antibodies overnight at 4°C: S100β antibody specifically labels Schwann cells, as validated in previous studies (11), rabbit anti-Iba1 (1:1000, 19741, Wako, Osaka, Japan), mouse anti-S100β (1:500, bsm-10832M, Bioss), and rabbit anti-Panx 1 (1:200, A00915-2, Boster, China), rabbit anti-IL-1β (1: 200, A00101, Boster, China), rabbit anti-TNF-α (1: 400, BA0131, Boster, China), rabbit anti-P2X7 (1:200, 28207-1-AP, proteintech, USA). After being submerged in PBS (PH7.4), the glass slides were shaken three times for five minutes each using the decolorization shaker. The tissues were covered with HRP secondary antibodies of the appropriate species of primary antibody in the ring after the sections had been slightly dried. They were then incubated for 50 minutes at room temperature in the dark before being rinsed three times with PBS. After ten to fifteen minutes of reaction with LL570 or LL520 fluorescent dye, three PBS washes were performed.

The images of the tissues were checked by 3D HISTECH (Pannoramic DESK, Hungary). The rings were treated with DAPI dye solution after the portions had been allowed to dry slightly. At room temperature, the slices were incubated for ten minutes. Three animals per group provided at least three non-overlapping sections from which all of the positive cells were counted.

### Schwann cell culture and LIPUS stimulation

Schwann cells were obtained from ATCC company (CRL-2766). Cells were used between passages 3–8 to ensure consistency and viability. Following a 24-hour incubation period in 3 mL serum-free DMEM containing 200 ng/mL TNF-α, either with or without LIPUS stimulation (continuous 2-day treatment, day 0 to day 1, 1.5MHz, 30mW/cm2, 20min/day), the cells were extracted for gene analysis. The cells were seeded in 6-well plates.

### RNA sequencing

**Figure 1:**
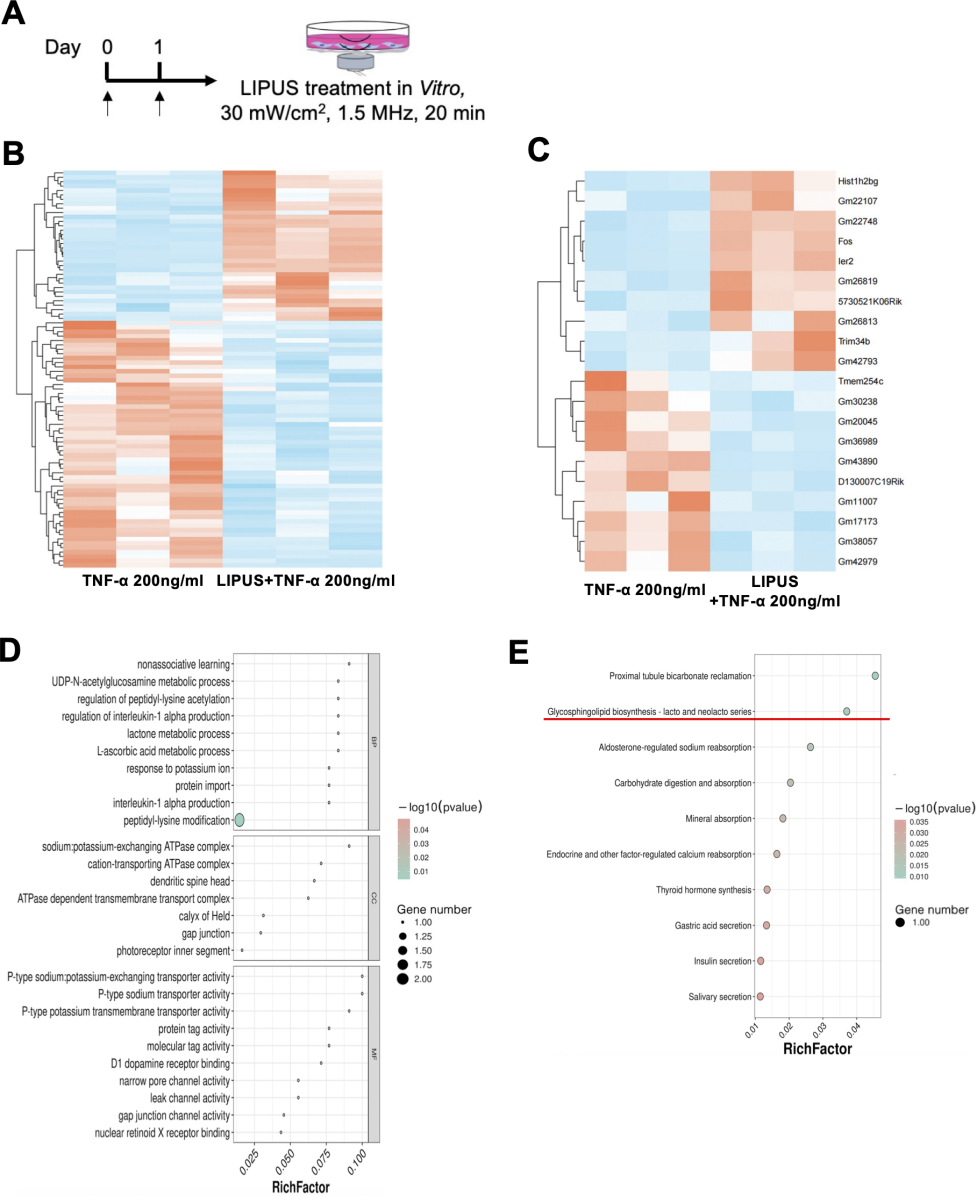
RNA-sequencing analysis of TNF-α-treated Schwann cells with or without LIPUS stimulation. **(A)** Time course of LIPUS treatment. **(B, C)** Heat map analysis. **(D)** GO analysis. **(E)** KEGG enrichment analysis (n=3, per group).

Three days post-pIONL surgery, total RNA was isolated from the ION using Invitrogen’s TRIzol reagent. Schwann cells were subjected to a 24-hour treatment with TNF-α at a concentration of 200ng/ml, either with or without LIPUS (a continuous 2-day treatment, from day 0 to day 1, at 1.5MHz, 30mW/cm^2^, for 20 minutes per day). The clean reads were then meticulously aligned using the HISAT2 software, resulting in a clear mapping to the mm10 mouse reference genome. This alignment enabled the accurate identification of both uniquely mapped and repeat reads (22). Differentially expressed genes were identified using edgeR with a false discovery rate (FDR) adjustment (Benjamini-Hochberg method). Genes with FDR < 0.05 and fold change >2 were considered significant,>. Additionally, we conducted gene set enrichment analysis to pinpoint overrepresented gene or protein categories within broader datasets, leveraging both Gene Ontology and KEGG pathway annotations (23, 24). The raw sequencing data were submitted to the NCBI Sequence Read Archive database under the accession number (PRJNA1357878 to **Supplementary Figure 1**, PRJNA1357852 to **Figure 1**).

### Sample size and reproducibility

All experiments were performed with at least three independent biological replicates. The exact value of n for each experiment is reported in the corresponding figure legends.

### Statistical analysis

In our pre test, sample sizes were determined based on previous studies and power analysis (α=0.05, power=0.8) using G*Power software. For behavioral tests, n=5 per group was sufficient to detect a 30% difference in pain thresholds. The statistical analysis involved comparing group means through Student’s t-test for pairwise evaluations, whereas Tukey’s *post hoc* test was utilized when examining multiple groups. For behavioral time-courses, we prespecified the area under the curve (AUC) as the primary endpoint. Longitudinal trajectories were compared using linear mixed-effects models (group × time; random intercept for mouse). All computations were performed using GraphPad Prism version 10 for accurate results. A p-value threshold of less than 0.05 was considered statistically significant.

## Results

### pIONL upregulated nocifensive behavior score and the expression of Panx 1 and pro-inflammatory cytokines

**Figure 2:**
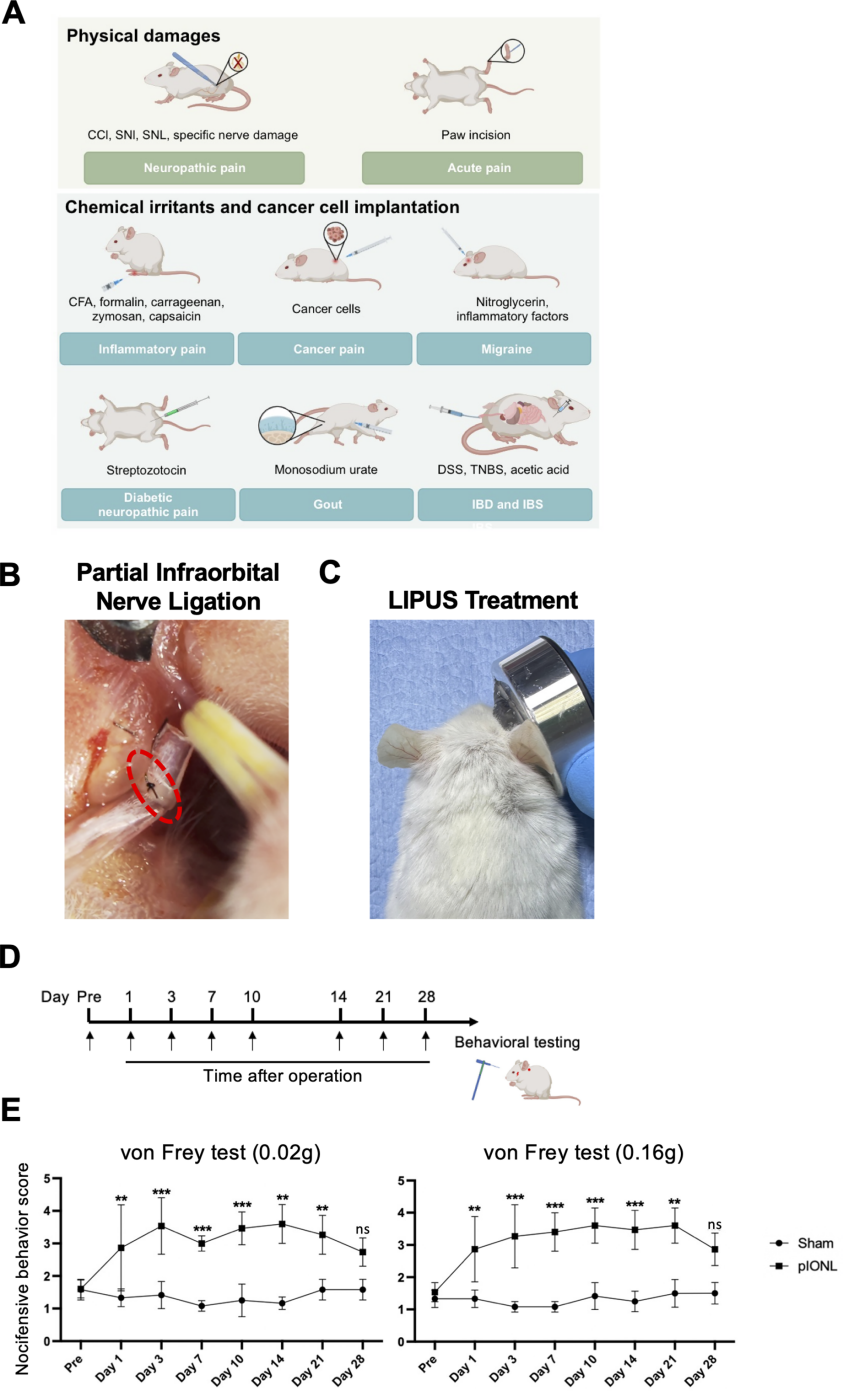
Partial infraorbital nerve ligation (pIONL)-induced trigeminal neuropathic pain (TNP). **(A)** Current models to induce pain. **(B)** Surgical image of the pIONL model in mice. **(C)** LIPUS treatment on the whisker pad of mice. LIPUS was immediately exposed to the injury side after pIONL (30mW/cm^2^, 20min) and continuously treat for 7 days. **(D, E)** Nocifensive behavior score of 0.02g and 0.16g filaments. Linear mixed-effects model (Group×Time; random intercept for mouse), with AUC_{0–28 d} prespecified as the primary endpoint; *post-hoc* Tukey where applicable (n=5/group). Mean ± SD shown., **p<0.01, ***p<0.001 (sham vs. pIONL).

There are several models to induce pain, in our studies we induced TNP by using pIONL model (**Figures 2A, B**). We applied LIPUS on the injury side of whisker pad after pIONL, results revealed that mechanical allodynia increased on day1 and maintained until day 21 after pIONL compared to sham group (**Figures 2C–E**). After pIONL, neuroinflammation modulates the pathological progress of TNP. To explore the inflammation condition in ION after pIONL surgery, we performed RNA-sequencing. Volcano plot showed pIONL group has 835 genes upregulated after injury compared to Sham. Also, the expression of Panx 1 is high in ION but not Panx 2 or Panx 3. Other inflammatory genes also upregulated after pIONL 3 days. According to the KEGG pathway enrichment analysis, there is a strong correlation between inflammation and pain since TNF NF-kappa B, the toll-like receptor signaling pathway, and the cytokine-cytokine receptor interaction are all strongly enriched (Additional file: **Supplementary Figure 1**).

### Local treatment with LIPUS ameliorated pIONL induced mechanical allodynia and neuroinflammation in ION

**Figure 3:**
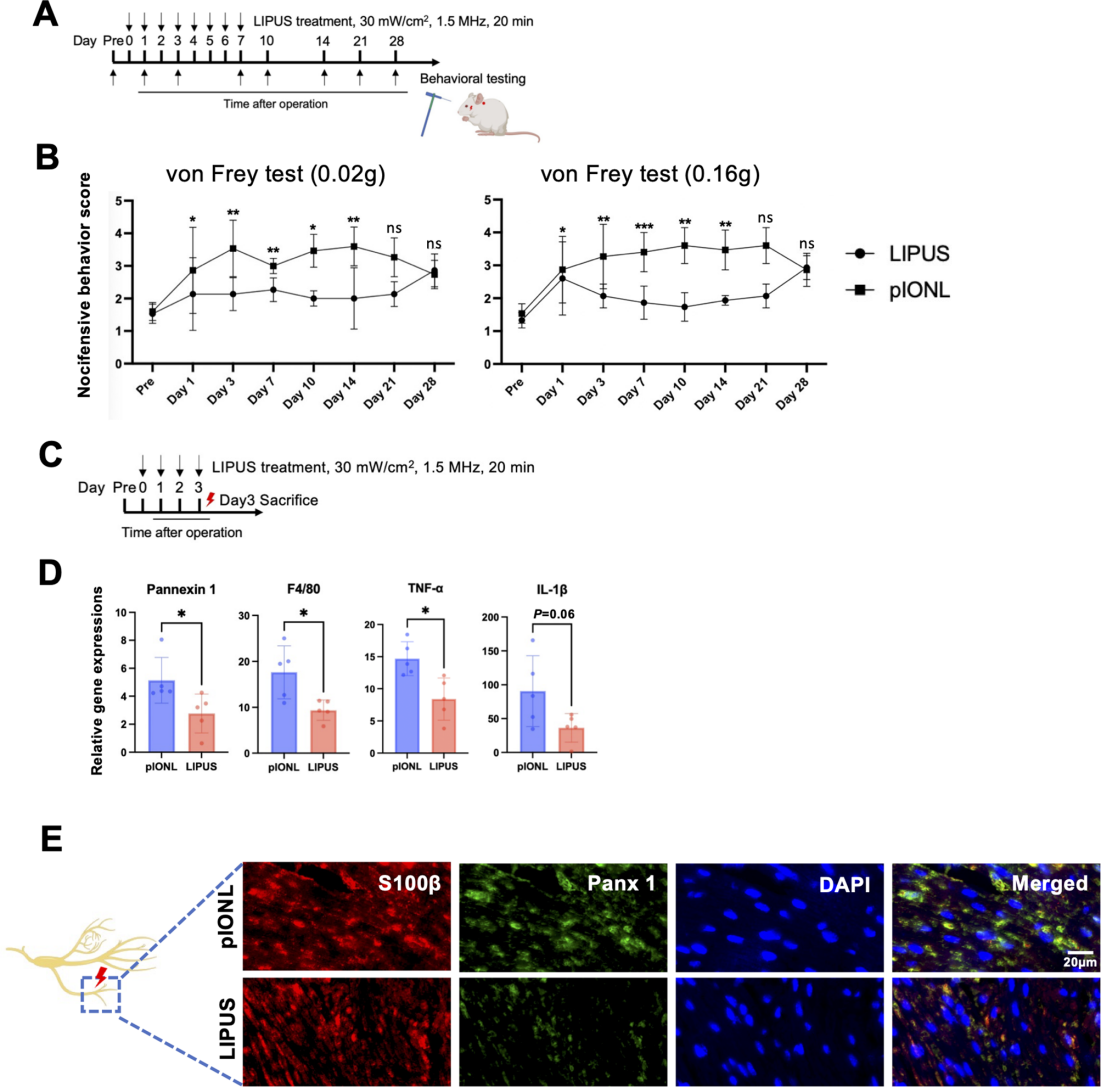
LIPUS treatment attenuates partial infraorbital nerve ligation (pIONL)-induced trigeminal neuropathic pain (TNP) and inflammation in infraorbital nerve (ION). **(A)** Duration and application schedule of LIPUS therapy. **(B)** Pain response measurements using 0.02g and 0.16g von Frey filaments. Statistical analysis performed via mixed-effects (Group×Time), primary ΔAUC {0–21 d} (95% CI) (n=5 per group), with results presented as mean ± SD. **(C)** Timeline of LIPUS intervention. **(D)** Quantitative PCR assessment of Panx 1, F4/80, TNF-α, and IL-1β mRNA levels in the infraorbital nerve three days post-pIONL. Student’s t-test (n=5 per group); data shown as mean ± SD. **(E)** Immunofluorescence staining of Panx 1 and S100β in the infraorbital nerve. Scale bar: 20μm. Analysis included at least three sections from three animals per group. Student’s t-test (n=3 per group); data expressed as mean ± SD. * p<0.05, ** p<0.01, *** p<0.001 (LIPUS vs. pIONL).

We locally treat LIPUS on the ipsilateral side of whisker pad immediately after pIONL, when TNP not yet developed. As shown in **Figures 3A, B**, continuous LIPUS treatment significantly inhibited pIONL-induced mechanical allodynia, and this efficacy lasted for at least 14 days. Day 3 was chosen to capture early inflammatory responses, while day 7 corresponds to peak microglial activation and pain maintenance in this model.

To test LIPUS treatment on the early phase of TNP, we examined the gene expression of Panx 1, F4/80, TNF-α, IL-1β in the ipsilateral ION on day3. As shown in **Figures 3C, D**, gene analysis revealed that LIPUS treatment decreased the expression of Panx 1, F4/80, TNF-α, compared to pIONL group. A trend toward reduction in IL-1β was observed (*p* = 0.06). Immunofluorescent staining data showed that LIPUS treatment significantly downregulated the expression of Panx 1/S100β compared with pIONL group (**Figure 3E**). Although our data has shown that LIPUS 30mW/cm^2^ induced M2-polarized macrophages after 24h exposure *in vitro* (**Additional file:****Supplementary Figure 2**), no effect was observed on inducing M2-polarized macrophages, CD206, arginase-1 (Arg-1) and Ym-1 after 3 days exposure *in vivo* (**Additional file:****Supplementary Figure 3**). We hypothesized that the effect of LIPUS on macrophages may take time *in vivo*. Additionally, our findings revealed no differences between the pIONL and LIPUS groups in terms of brain derived neurotrophic factor (BDNF), nerve growth factors (NGF), glial derived neurotrophic factors (GDNF), and TGF-β (**Additional file:****Supplementary Figure 4**), which have neurotrophic effect on Schwann cells. These findings showed that the LIPUS converted the pro-inflammatory microenvironment of pIONL possibly because of inhibition of the activation of Schwann cells.

### LIPUS suppressed pro-inflammatory cytokines in TG and suppressed microglial activation in spinal cord after pIONL

**Figure 4:**
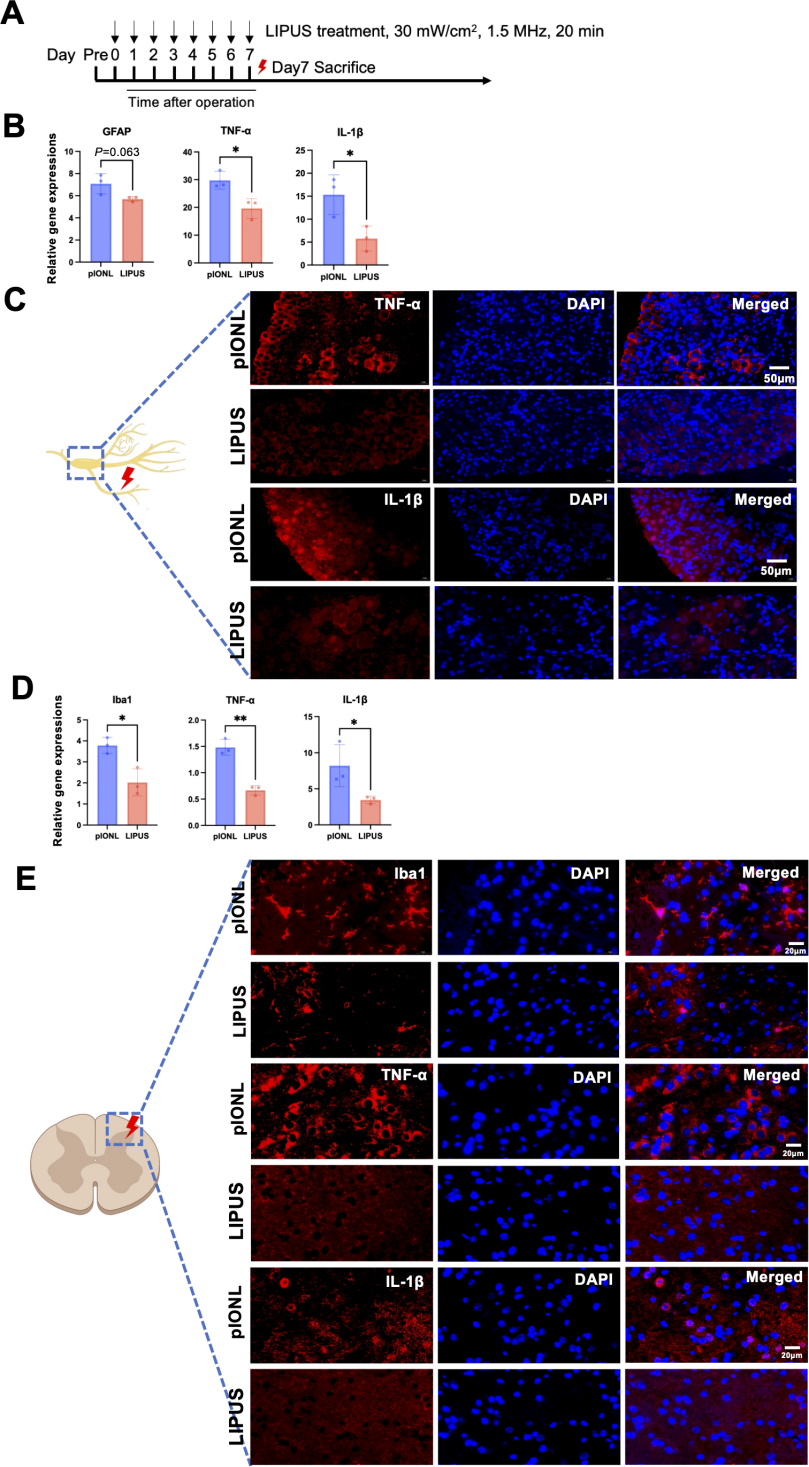
LIPUS treatment attenuates inflammatory markers in trigeminal ganglion and spinal cord. **(A)** Experimental timeline for LIPUS administration. **(B)** Quantitative PCR (qPCR) analysis of GFAP, TNF-α, and IL-1β mRNA levels in the trigeminal ganglion (TG) seven days post-pIONL (Student’s t-test, n=3 per group). **(C)** Immunofluorescence staining of TNF-α and IL-1β in TG (scale bar: 50 μm). **(D)** qPCR assessment of Iba1, TNF-α, and IL-1β mRNA expression in the spinal cord seven days post-pIONL (Student’s t-test, n=3 per group). **(E)** Immunofluorescence images of Iba1, TNF-α, and IL-1β in spinal cord tissue (scale bar: 20 μm). Analysis included at least three sections from three animals per group. Data presented as mean ± SD. *p < 0.05, **p < 0.01 (pIONL vs. LIPUS).

In previous study, it has been investigated that microgliosis happened in spinal cord after pIONL surgery, which sustained the pain. The mRNA expression of genes related to GFAP, Iba1, and pro-inflammatory cytokines in TG and spinal cord on day 7 following pIONL surgery was examined in order to investigate the specific state of the microenvironment. GFAP expression in TG, indicating satellite glial cell activation, was increased after pIONL and reduced by LIPUS, supporting its role in modulating glial-neuronal interactions. TG had elevated expressions of inflammatory genes, including GFAP, TNF-α, and IL-1β, which were reduced by LIPUS therapy (**Figures 4A-C**). Also, LIPUS downregulated expressions of Iba1, IL-1β and TNF-α in injured C1-C2 spinal cord (**Figure 4D**). Immunofluorescent staining revealed the phenomenon of microgliosis after pIONL while LIPUS treatment significantly decreased the number of Iba1^+^ microglia (**Figure 4E**).

### LIPUS suppresses Panx 1, alleviating pIONL-triggered pain sensitivity and inflammatory response

**Figure 5:**
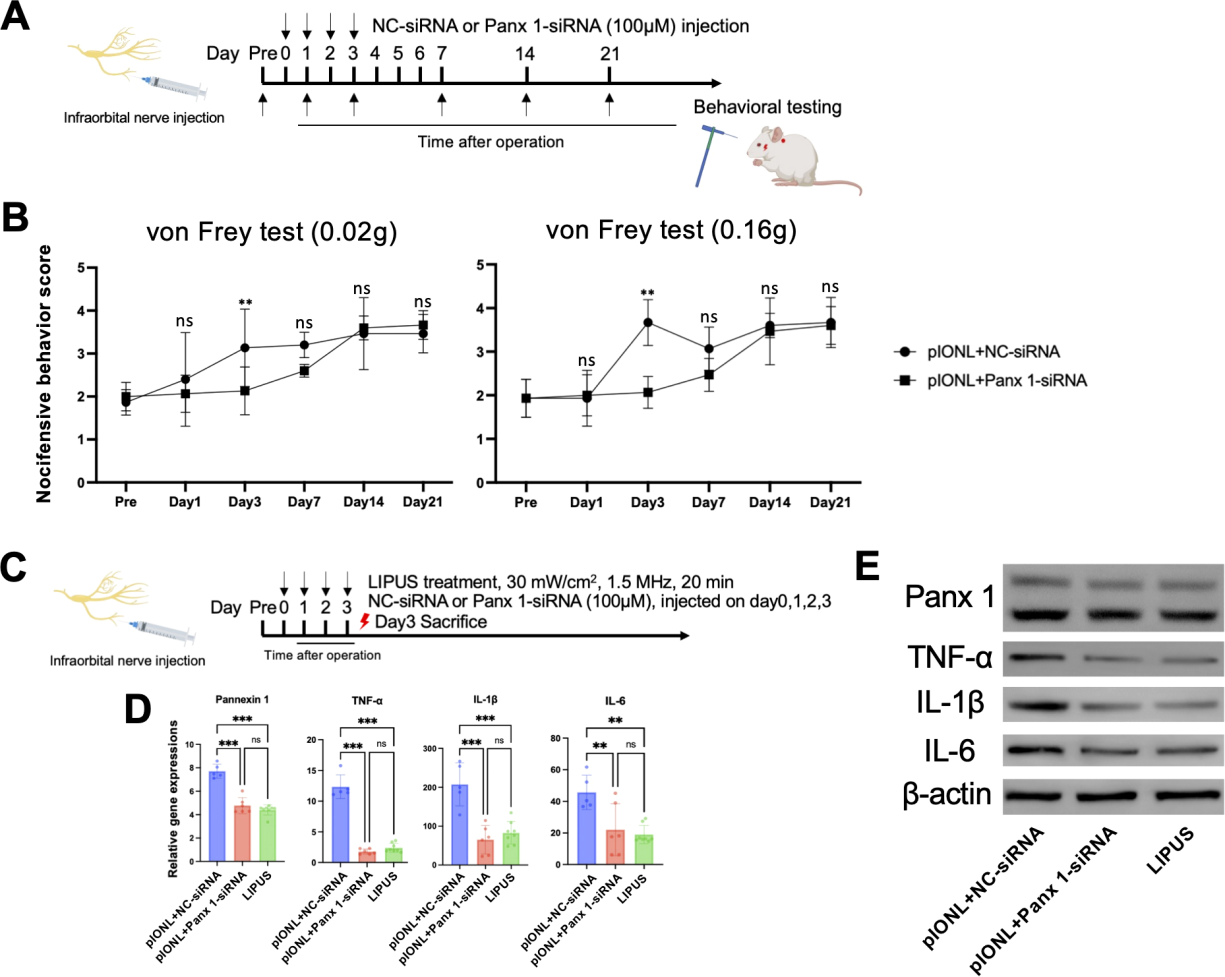
Panx1-siRNA reverses pain-related behavior. **(A)** Schedule of siRNA injections. **(B)** Panx1-siRNA administration significantly reduced pain sensitivity following partial infraorbital nerve ligation (pIONL) (mixed-effects (Group×Time); primary ΔAUC_{0–7 d} (95% CI), n=5 per group; mean ± SD). **(C)** Combined timeline of siRNA injections and LIPUS therapy. **(D, E)** LIPUS and Panx1-siRNA suppressed Panx1, TNF-α, and IL-1β expression in the infraorbital nerve at both transcriptional (n=5–8 per group) and translational levels (n=3 per group) (Student’s t-test; mean ± SD). **p < 0.01, ***p < 0.001 (pIONL+NC-siRNA vs. pIONL+Panx1-siRNA).

Next, to check the analgesic efficacy of LIPUS by inhibiting Panx 1, we compared mechanical allodynia after NC-siRNA and Panx 1- siRNA intra-ION injection, respectively. We treated mice with NC-siRNA or Panx 1-siRNA immediately after pIONL surgery. At day 1 post-pIONL, we observed that there were no variations in the TNP thresholds across the groups. On the third day, however, Panx 1-siRNA reversed TNP.pIONL containing the NC-siRNA group did not exhibit any analgesic effects. The 0.16g filament produced the same outcome as the 0.02g filament (**Figures 5A, B**), indicating that Panx 1 inhibition is adequate to reduce pIONL-induced TNP.

To delve deeper into the gene expressions resulting from treatment with NC-siRNA or Panx 1 siRNA, we observed the expressions of Panx 1 and pro-inflammatory factors in the ION three days post-siRNA or LIPUS administration. Comparing pIONL with the NC-siRNA group to pIONL with the Panx 1-siRNA group, we discovered that the LIPUS group nearly reduced Panx 1 expression by 50%. Additionally, the IL-6, TNF-α and IL-1β gene expressions in these groups exhibited the same downward trend (**Figures 5C, D**). At the protein level, we saw the same pattern of Panx 1, IL-6, TNF-α, IL-1β in these groups (**Figure 5E**). According to these findings, LIPUS is adequate to reduce pIONL-induced TNP via Panx 1.

### LIPUS inhibited Schwann cell Panx 1 and suppressed pro-inflammatory activities *in Vitro*

**Figure 6:**
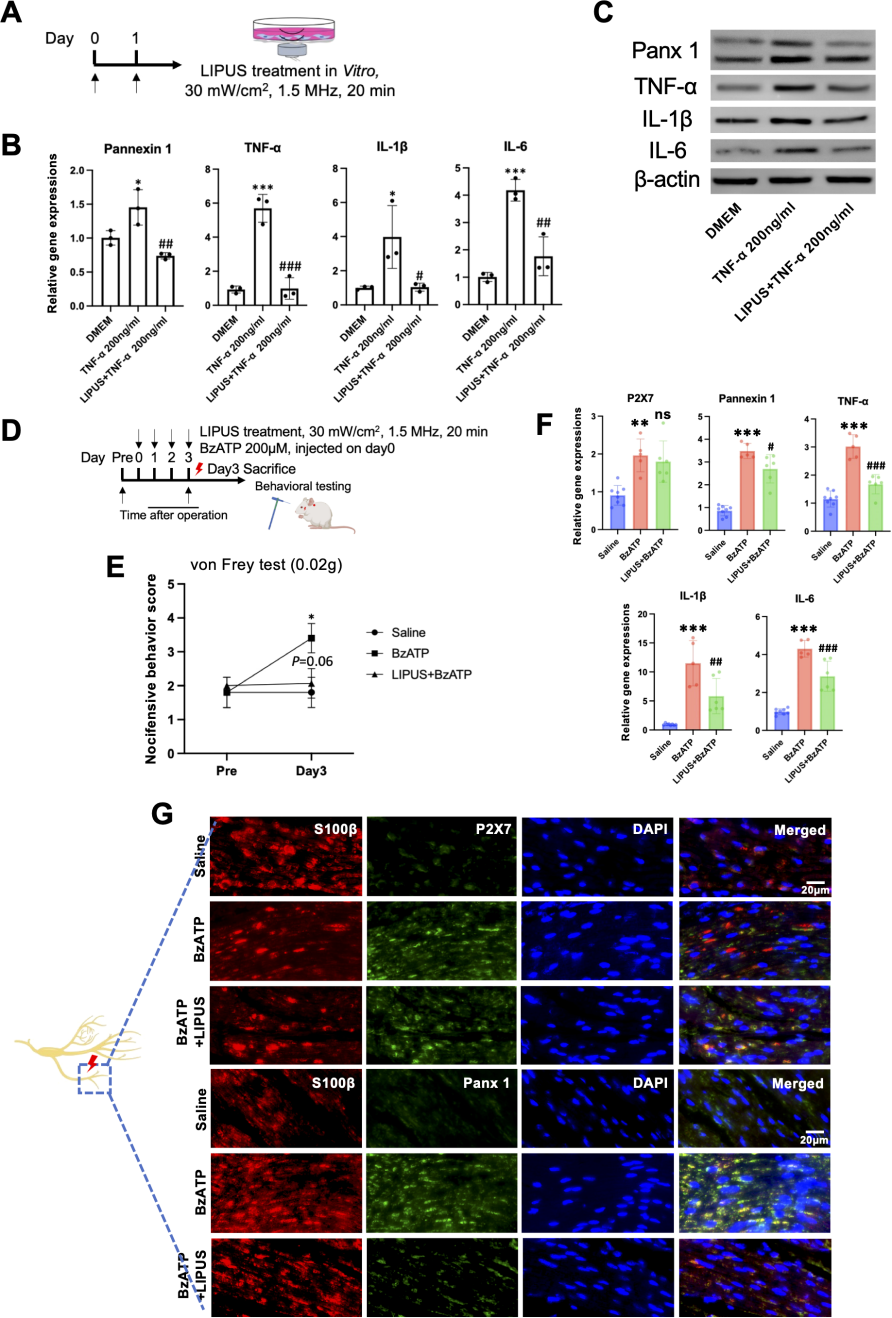
Mechanistic validation of LIPUS efficacy in vivo and *in vitro*. **(A)** LIPUS treatment protocol. **(B)** RT-qPCR of TNF-α-stimulated Schwann cells revealed LIPUS-mediated suppression of pro-nociceptive channels and inflammatory factors (Tukey’s test, n=3 per group; mean ± SD). *p < 0.05, ***p < 0.001 (TNF-α vs. DMEM); #p < 0.05, ##p < 0.01, ###p < 0.001 (TNF-α vs. LIPUS). **(C)** Western blot analysis of Panx1, TNF-α, IL-1β, and IL-6 protein expression (n=3 per group). **(D)** Timeline for BzATP injections and LIPUS intervention. **(E)** Behavioral pain scores in response to 0.02g filament stimulation (statistics as in **Figure 2** with primary ΔAUC_{0–3 d} (95% CI), n=5 per group; mean ± SD). **(F)** RT-qPCR of BzATP-treated infraorbital nerve with/without LIPUS (day 3; Tukey’s test, n=5–8 per group; mean ± SD). *p < 0.05, **p < 0.01, ***p < 0.001 (BzATP vs. saline); #p < 0.05, ##p < 0.01, ###p < 0.001, n.s. (BzATP vs. LIPUS+BzATP). **(G)** Immunofluorescence co-staining of S100β/P2X7 and S100β/Panx1 in infraorbital nerve (scale bar: 20 μm). Analysis included at least three sections from three animals per group.

Previous study has shown that Panx 1 participated in inflammatory activities in Schwann cells (11). We explored LIPUS exposure in cultured Schwann cells, which may further confirm the anti-inflammatory function of LIPUS. We used TNF-α to create an inflammatory environment. TNF-α-exposed Schwann cells (0 and 200ng/ml) were incubated for 24 hours at 37˚C. The TNF-α (200ng/ml) group exhibited increased mRNA levels of Panx 1, TNF-α, IL-6 and IL-1β. In contrast, LIPUS induced approximate 50% downregulation of Panx 1 and IL-6, and approximate 80% downregulation of TNF-α and IL-1β (**Figures 6A, B**). We also observed the same tendency of Panx 1, TNF-α, IL-6 and IL-1β in these groups at protein levels (**Figure 6C**). These findings suggest that LIPUS perform as the role as Panx 1 blocker and contribute to the inhibition of inflammatory activities in Schwann cells.

### LIPUS suppressed Panx 1 and pro-inflammatory activities by ION injected BzATP *in Vivo*

Numerous P2X7 receptor was observed in Schwann cells (25). Research highlights the pivotal function of Panx 1’s engagement with the P2X7 receptor in both the peripheral and central nervous systems. P2X7 was considered as the key candidate to interplay with Panx 1 (26–28). To determine if LIPUS directly inhibits Panx1, we injected BzATP (200 μM) into the ION to activate Panx1 channels (29),,. For LIPUS group, we performed LIPUS immediately after BzATP injection for continuous 3 days (30mW/cm^2^, 20min).

von Frey test (0.02g) showed that BzATP induced TNP compared to saline group while LIPUS reversed BzATP induced mechanical allodynia (**Figures 6D, E**). The mRNA expression of P2X7, Panx 1, TNF-α, IL-6 and IL-1β were upregulated after BzATP injection compared with saline group while LIPUS downregulated the expressions of these markers (**Figure 6F**) on day3. Immunofluorescent staining showed the same data as gene analysis (**Figure 6G**). These results indicate that activation of Panx 1 related to P2X7, however, at least no therapeutic effect of LIPUS treatment was observed on Schwann cell P2X7 on day3 in *Vivo* study.

### LIPUS downregulates potential genes and pathways in Schwann cell *in Vitro*

Finally, to investigate the potential mechanism of LIPUS treated Schwann cells in TNP, we performed RNA-sequencing by using TNF-α (200ng/ml) and LIPUS treated Schwann cells. Our results found that LIPUS upregulates genes, such as hist1h2bg, gm22107, gm22748, fos, ler2 and so on, while downregulates genes, such as tmem254c, gm30238, gm20045 and so on (**Figures 1A–C**). Also, GO and KEGG analysis showed that LIPUS downregulated inflammation related pathways, interleukin-1 alpha production; ion channel related pathways, sodium: potassium-exchanging ATPase complex; glycosphingolipid biosynthesis-lacto and neolacto series and gap junction and so on (**Figures 1D, E**).

## Discussion

**Figure 7:**
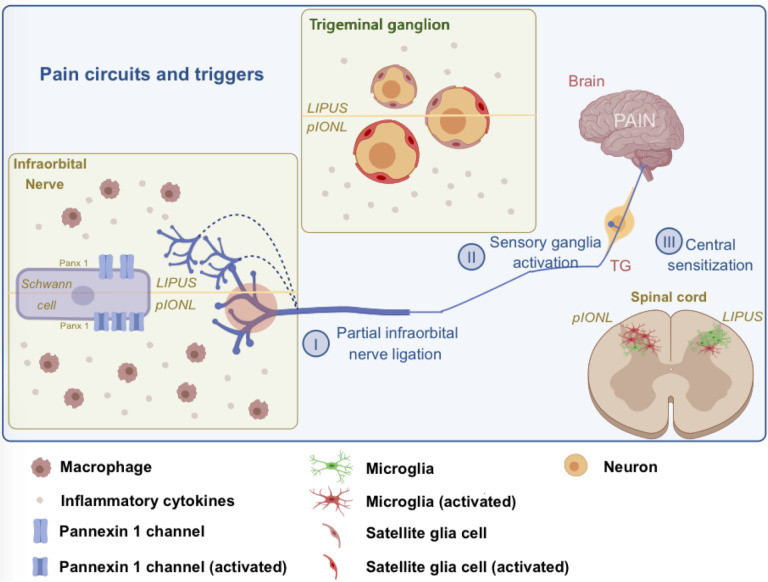
Cellular and molecular mechanisms inducing to Panx 1-mediated neuroinflammation and mechanical allodynia in trigeminal neuropathic pain (TNP) model. pIONL induces activation of Panx 1, secretion of pro-inflammatory cytokines in Schwann cell and accumulation of macrophages. Panx 1 activation in Schwann cell produces the satellite glial cells activation and pro-inflammatory cytokines secretion in trigeminal ganglion (TG). The crosstalk between satellite glial cells and neurons sustains TNP. Sequence of inflammatory responses in infraorbital nerve and TG induces microglia activation in spinal cord. LIPUS treatment inhibits Schwann cell Panx 1 and suppress the continuous inflammation responses, which reverses the inflammatory microenvironment in peripheral system and central nervous system and finally relieve the TNP.

Increasing evidence has revealed that LIPUS consistent exposure alleviates several diseases (14–16). However, the mechanism of the potential effect of LIPUS on TNP remains to be unknown. Our study has demonstrated the effectiveness of LIPUS on pIONL-induced TNP model through regulate the Schwann cells Panx 1. First, pIONL induced a persistent pain (>21 days) and the upregulation of Panx 1 within ION on day3. Second, local treatment of LIPUS on the whisker pad of mice effectively reduced pIONL-induced mechanical allodynia and reduced neuroinflammation not only in partial ION, ipsilateral TG but also in the injured dorsal horn of spinal cord. Third, *in vivo* research showed that Panx 1 inhibition using LIPUS or Panx 1-siRNA could suppress the production of inflammatory factors. Forth, TNF-α increased the expression of Panx 1 and inflammatory mediators *in vitro* study while LIPUS inhibited those expressions. Finally, BzATP activated Panx 1 and induced TNP *in vivo* while LIPUS ameliorated BzATP induced pain. Our findings firstly demonstrate that LIPUS inhibited Schwann cell Panx 1 and hence ameliorated the neuroinflammation not only in TG but also in spinal cord (**Figure 7**).

LIPUS produces vibrational forces that increase blood flow and reduce discomfort (14, 15, 30–35),. Attenuation is minimal (<5%) with gaps <4 mm, making ION treatment feasible. LIPUS efficacy was previously shown in a sciatic nerve ligation model (36), supporting its potential for pIONL-mediated TNP.

Neuroinflammation is related to several neurological disorders (20, 37–39). Schwann cells derived from neural crest cells, which responsible for forming the myelin sheath of the trigeminal nerve (40). Schwann cells promote nerve regeneration and repair and produce several cytokines that provide nourishment to peripheral nerve axons (41–43). Also, when injury occurs, Schwann cells act as the first immune response to nerve injury (44). A recent investigation has reported that LIPUS promoted Schwann cells proliferation and viability via GSK-3β/β-catenin signaling pathway. Much attention on Schwann cells was on its ability of nerve regeneration (45–47). Our data showed that LIPUS significantly ameliorated mechanical allodynia in pIONL mice. LIPUS show the anti-nociceptive effect on day1 after pIONL and lasted for 14 days. The gradual decrease in mechanical allodynia in pIONL groups over time may reflect partial nerve adaptation or compensatory mechanisms. However, LIPUS treatment significantly accelerated this recovery. In the CNS, pIONL induced microgliosis and pro-inflammatory cytokines imply the maintenance of TNP; meanwhile, LIPUS treatment restored the pain. To further confirm the crucial role of LIPUS in reducing neuroinflammation, we directly examined the gene expressions of Panx 1, F4/80, TNF-α and IL-1β in ION. Although we found LIPUS may induce M2-polarized macrophages in a partial sciatic nerve ligation model in mice, there is no difference of CD206, Arg-1 and Ym-1 between pIONL group and LIPUS group on day3 after pIONL. Also, our data showed the different parameters of LIPUS stimulation induced increased expression of CD206, GDNF and decreased the expression of TNF-α, IL-β in RAW264.7 especially in parameter of 30mW/cm^2^. A previous study showed that exosomes derived from HUVECs alleviated inflammation in neural cells (48). however, LIPUS is much safer compared to exosomes or stem cells. Our data suggested that LIPUS treatment prevented TNP mainly through the inhibition of activated Schwann cells after nerve injury. The sustained effect may be due to long-term changes in glial activation or epigenetic modifications, as suggested by RNA-seq data.

Transmission of orofacial pain signals via the TG. Activated satellite glial cells expressing GFAP and secreted pro-inflammatory cytokines (3). LIPUS treatment decreased the expressions of GFAP, TNF-α and IL-1β in TG compared to pIONL group, which means LIPUS shut down the crosstalk between satellite glial cells and neurons. Microglia are resident immune cells in CNS, which play a role as an immune defender (49–51). Our study has demonstrated the activated microglia played an amoeboid or hypertrophied morphology which was different from the ramified morphology in the sham group and LIPUS group. Furthermore, LIPUS treatment downregulated the expression of the microglial marker Iba-1 and TNF-α and IL-1β in spinal cord following pIONL. The TG is in a depression of the temporal bone and C1-C2 spinal cord is in C1 and C2 vertebrae. Also, the capability of LIPUS is hard to pass through the bone. Taken together, LIPUS restored the inflammatory microenvironment in TG and spinal cord mainly relied on inhibiting activated Schwann cells located in ION.

Upon activation, Panx 1 channel release ATP as well as cytokines (52, 53). Panx 1 plays a crucial role in neuropathic pain, which is broadly expressed within immune cells, glial cells and neurons (8, 9, 54–57). In central post-stroke pain, blocking of microglial Panx 1 channels reduced neural damage mediated by the inflammatory response (58). It also has been shown that Panx 1 channel activation related to spinal central sensitization process (54, 59). Emerging research indicates a link between GFAP-positive glial and neuronal Panx 1 expression and varying stages of hypersensitivity within an orofacial pain model (60). Inhibiting Panx 1 in neurons, glia, and immune cells alleviates neuropathic pain. Schwann cells are the first response for nerve injury; however, the exhaustive mechanism is still unknow. Previous study has reported that Schwann cell TRPA1 mediated first inflammatory response in CCI model in mice (61). A recent study also investigated the role of Schwann cell Panx 1, which is related to inflammation modulation (11). Since no Panx 1 agonist was found, we further confirm the analgesic effect of LIPUS by comparing with Panx 1-siRNA. Results indicated that Panx1 inhibition via LIPUS therapy reduced TNP and Panx1-siRNA effects *in vivo*, which indicated the analgesic effect of LIPUS treatment. Schwann cells highly express TLR2, TLR3, and TLR4 (62). TNF-α exposure upregulated Panx 1, TNF-α, IL-6 and IL-1β levels, whereas LIPUS counteracted these effects. Our tests in living organisms and lab cultures confirmed that both Panx1-siRNA and LIPUS treatments significantly suppressed Panx1 expression while simultaneously reducing levels of TNF-α, IL-1β, and IL-6. As a key player in immune responses, tumor necrosis factor alpha (TNF-α) plays a pivotal role in mediating neuro-inflammatory signaling pathways (63). A recent study demonstrated TNF-α mediated RIPK1 pathway, which participates in the TNP (64). IL-1β is also involved in the neuropathic pain associated with inflammation (6, 65). It also has been reported that IL-6 participates in neuropathic pain related to nerve trauma, cancer, and inflammation (66–68). In the future, we may construct the vector to overexpress Panx 1 to further confirm the effect of LIPUS.

Recent studies have shown that P2X7 interplays with Panx 1 in peripheral nervous system and CNS (26–28). We further investigated whether LIPUS inhibited Panx 1 through P2X7. BzATP (200μm), which is the agonist of P2X7, was intra-ION injected. BzATP increased P2X7, Panx 1, TNF-α, IL-6 and IL-1β levels by day 3. LIPUS reduced only Panx 1, TNF-α, IL-1β, and IL-6 expression at the same timepoint. These results showed that activation of P2X7 receptor increased the expression of Panx 1, however, the analgesic effect of LIPUS relies on suppressing Panx 1. Our results together with these findings suggested that LIPUS can be considered as a novel and potential candidate in ameliorating TNP through inhibiting Schwann cell Panx 1.

The production of IL-1α is usually driven by the nuclear factor (NF-kB) signaling pathway and inflammasome pathway activated by cells after injury or pathological factor stimulation. Research has shown that inflammatory factors such as TNF-α and bacterial lipopolysaccharide (LPS) can significantly induce upregulation of IL-1α (69, 70). In addition, the production of IL-1α may also have a positive feedback mechanism, which can amplify local inflammatory responses through its own or similar cytokine actions (71). IL-1α is involved in occurrence of neuropathic pain and neuroinflammation, and its effects directly or indirectly affect the function of primary sensory neurons (3, 72, 73). Researchers have found that in some chronic inflammations, the expression level of IL-1α is significantly increased (74). More importantly, this cytokine also activates multiple signaling pathways involved in pain signal transmission by binding to its specific receptors (75, 76). The main function of sodium potassium exchange ATPase is to remove sodium ions from cells and introduce potassium ions through ATP driven mechanisms, thereby maintaining the cell’s transmembrane potential and normal ion concentration gradient (77). Sodium ion channels (Nav), especially specific subtypes Nav1.7, play an important role in TNP. The literature suggests that Nav1.7 is closely related to abnormal pain perception (78). Nav1.7 is known as the ‘low threshold sodium channel’, and its overexpression is associated with abnormal neuronal firing, which not only exacerbates pain symptoms but may also expand the range of pain perception through signal amplification (79). Downregulation of sodium-potassium ATPase and gap junction pathways may contribute to reduced neuronal excitability and pain signal propagation.

Gap junction channels are the bridge for signal transmission between neurons and glial cells, playing a crucial role in the amplification and propagation of pain signals. Connexin 43 (Cx43) is particularly significant in the inflammatory mechanism of TNP. Research has shown that the expression of Cx43 is abnormally increased in satellite glial cells (SGCs), and this increase is closely related to the upregulation of sodium channel Nav1.7, thereby regulating the amplification of pain signals (80). Cx36, another key gap junction protein, is highly expressed in the TG and is associated with increased pain induced by cold stimulation. Research has found that inhibitors targeting Cx36 can significantly alleviate cold hypersensitivity and regulate downstream signaling molecules (81). Leak channels are the core channels responsible for background ion flow and play an important role in maintaining neuronal potential stability. In recent years, studies have shown that two pore potassium channels (such as TREK1, TRESK, and TREK2) have potential roles in regulating neuronal resting potentials and combating neuropathic pain (82). For example, the loss or downregulation of TREK2 function may lead to neuronal overexcitation, which is associated with worsening pain. Kir4.1, which belongs to the same potassium ion channel, is mainly distributed in astrocytes and satellite glial cells. Its reduced function has been shown to induce pain behavior without direct nerve damage (83).

Lactone metabolism is typically associated with various biochemical reactions and drug metabolism. Lactone metabolism may influence pain through inflammatory mediator production, such as prostaglandins, which sensitize nociceptors. However, its direct role in TNP requires further investigation. Although the specific contribution of lactone metabolism in TNP is still unclear, it is indirectly related to the pain process by participating in the generation of inflammatory mediators. Many metabolites, such as prostaglandin E2, amplify the role of inflammatory signals by interacting with specific pain receptors, such as Nav1.7, thereby exacerbating pain (84). The global level changes of histone H3K4 monomethylation (H3K4me1) and H3K9 trimethylation (H3K9me3) are closely related to neuroinflammation and pain sensitization. Research has shown that in the NP model induced by pertussis toxin (PTX), the methylation level of spinal cord histones is significantly increased, and the combination of ultra-low dose naloxone and morphine can alleviate thermal hyperalgesia by downregulating H3K4me1 and H3K9me3 (85). In addition, oxidative stress caused by aluminum overload reduces the expression of BDNF by inhibiting the acetylation of histones H3K9 and H4K12, leading to cognitive dysfunction (86). These findings together suggest that peptidyl lysine modification is involved in the persistence of neuroinflammation and pain signals by regulating epigenetic reprogramming. Our research indicates that LIPUS downregulates all the above channels, suggesting that LIPUS plays an important role in multiple pathways in alleviating TNP.

KEGG pathway analysis showed significant downregulation of the Glycosylgolipid biosynthesis lacto and neolacto series. Glycosphingolipids (GSL) are a class of complex lipid molecules widely distributed on eukaryotic cell membranes, playing multiple key roles in many biological processes, including cell recognition, signal transduction, and membrane structure maintenance. The biosynthetic pathways of GSL can be divided into lacto-se series and neolacto-series (87). Panx 1 mainly induces intercellular signaling by releasing ATP, while GSL play a synergistic role in affecting the activity of P2X and P2Y receptors. GSL can act as signaling molecules in inflammatory cells, regulating downstream signaling pathways activated by exogenous ATP (88–90). The structural and functional characteristics of GSL determine their important role in neural signal transmission and inflammation regulation. Further experimental verification is needed to clarify the potential mechanism between above pathways and Panx 1, to investigate the pathology of TNP as well as develop the novel method to cure TNP.

### Limitations

This study has several limitations. First, the temporal resolution of LIPUS effects was limited to selected time points, future studies should include more time points to capture dynamic changes. Second, *vivo* experiments involved mixed cell populations, and we did not use cell-specific knockout models to confirm Schwann cell-specific mechanisms. Third, we only assessed immediate LIPUS treatment, future studies should evaluate delayed interventions to mimic clinical scenarios. Forth, our findings are based on the pIONL model, other TNP models (e.g., CCI) should be verified in future studies. Fifth, as TNP incidence is higher in women, future studies should include female subjects. Also, we did not include a LIPUS-only control group, thus, potential effects on normal tissues cannot be excluded. Finally, we focused on pro-inflammatory cytokines, anti-inflammatory factors (e.g., IL-10) should be assessed.

## Conclusion

In summary, we demonstrate the analgesic effect of LIPUS through inhibition of Schwann cell Panx 1. The present research confirmed that LIPUS reduced the production of TNF-α, IL-1β and IL-6 by inhibiting Panx 1 channel both *in Vivo* and *in Vitro* studies. Also, LIPUS downregulates several pathways in Schwann cells, which can be potential research target in TNP in the near future. Our study strongly proved that LIPUS is an effective and non-invasive treatment option for pIONL-induced TNP.

## Data Availability

The datasets presented in this study can be found in online repositories. The names of the repository/repositories and accession number(s) can be found in the article/**Supplementary Material**.
