# Supplementary material for: Low-intensity pulsed ultrasound ameliorates partial infraorbital nerve ligation-induced trigeminal neuropathic pain through inhibiting Schwann cell Pannexin 1 channel
Source: Front Immunol. 2026 Jan 5;16:1712759. doi: 10.3389/fimmu.2025.1712759 (PMC12812623; doi:10.3389/fimmu.2025.1712759)
Supplement: Supplementary file 1 [file DataSheet1.pdf]

Supplemental Figure 1

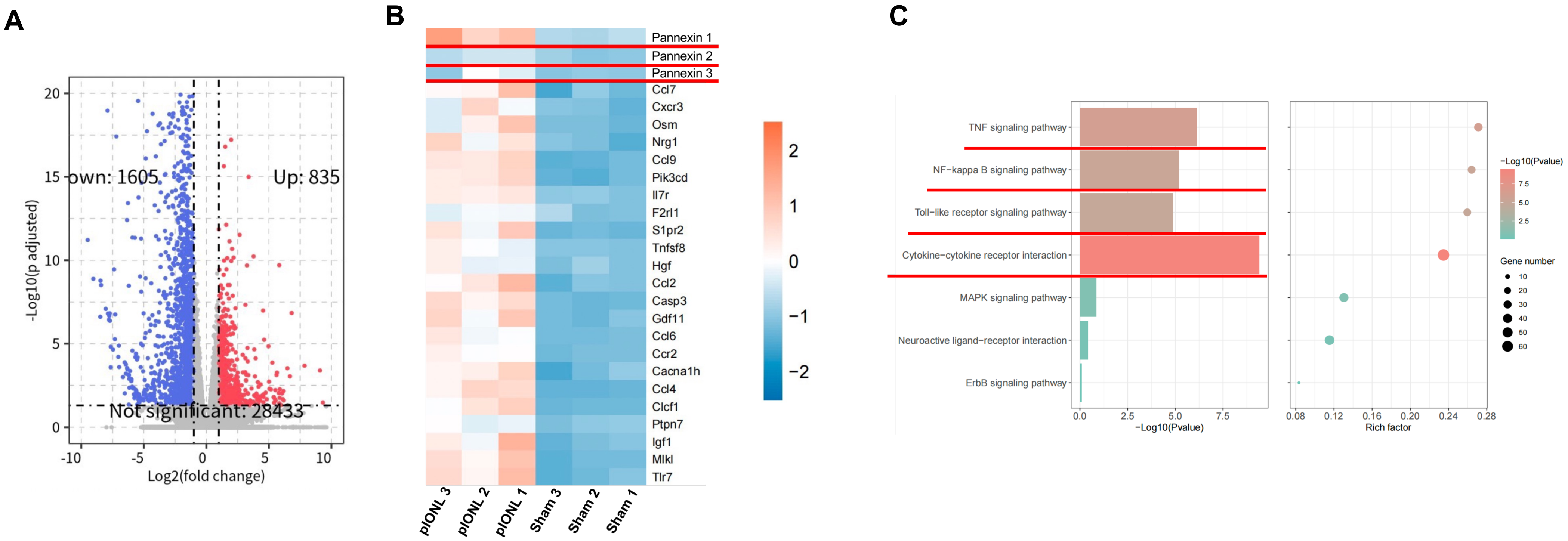

Supplemental Figure 1: RNA-sequencing analysis of ION after pIONL on day3. (**A**) Volcano plot of Sham vs pIONL. (**B**) Heat map analysis. (**C**) KEGG enrichment analysis (n=3, per group).

Supplemental Figure 2

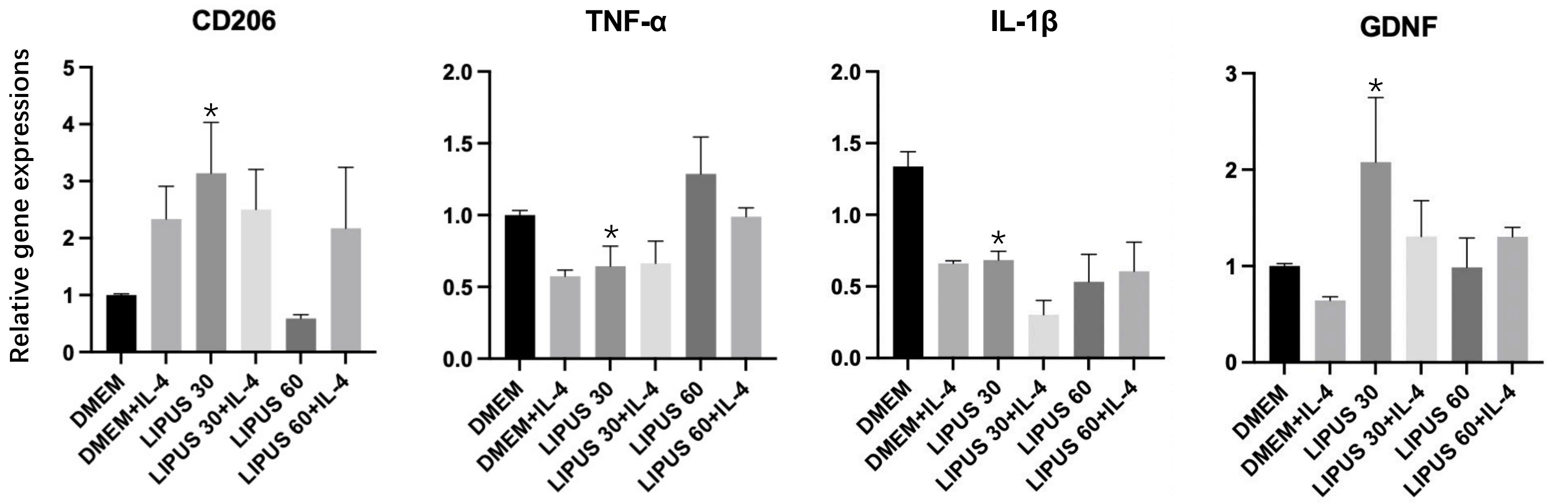

Supplemental Figure 2: The effect of LIPUS with different parameters on Raw264.7. LIPUS (continuous 2 days treatment, 1.5MHz, 20min/day) was exposed to Raw 264.7 cultured in 6-well dishes after changing to serum-free conditioned medium. qPCR analysis of M2 polarized macrophage marker, pro-inflammatory cytokines and neurotrophic factor after 24h. Tukey's multiple comparisons of means (n=3, per group). Data represent the mean ± SD. * $p < 0.05$ for LIPUS 30 vs. DMEM.

Supplemental Figure 3

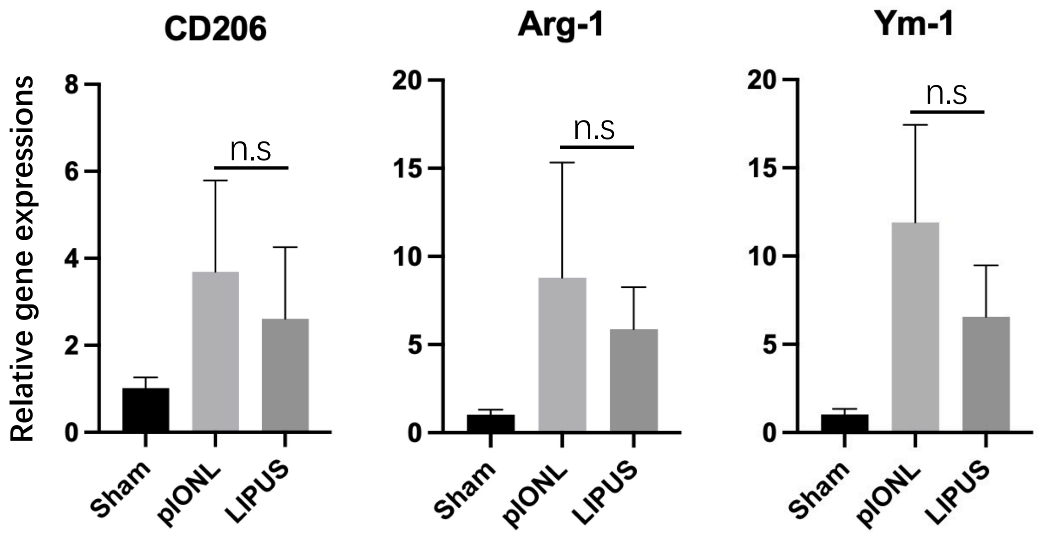

Supplemental Figure 3: LIPUS treatment had no effect on inducing M2-polarized macrophages in ION in mice post pIONL 3 days. Tukey's multiple comparisons of means (n=3, per group). Data represent the mean ± SD. n.s for pIONL vs. LIPUS.

Supplemental Figure 4

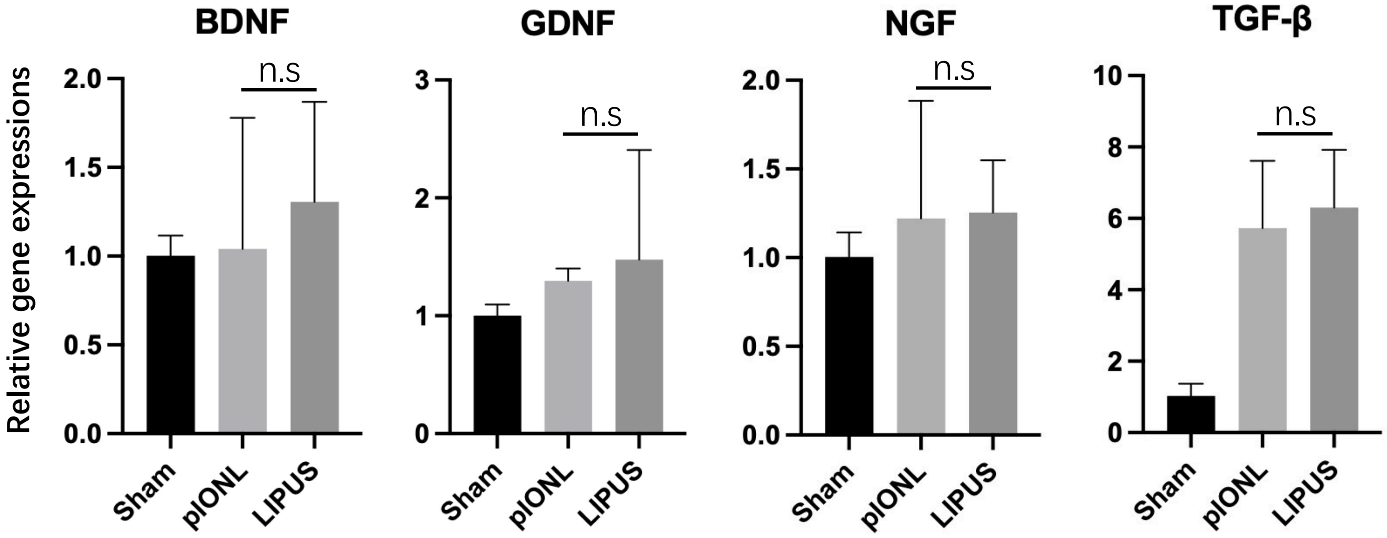

Supplemental Figure 4: : LIPUS treatment had no effect on inducing neurotrophic factors in ION in mice post pIONL 3 days. Tukey's multiple comparisons of means (n=3, per group). Data represent the mean ± SD. n.s for pIONL vs. LIPUS.
